# Attentional Bias Modification in Virtual Reality – A VR-Based Dot-Probe Task With 2D and 3D Stimuli

**DOI:** 10.3389/fpsyg.2019.02526

**Published:** 2019-11-13

**Authors:** Lichen Ma, Anne-Wil Kruijt, Sofia Nöjd, Elin Zetterlund, Gerhard Andersson, Per Carlbring

**Affiliations:** ^1^Department of Psychology, Stockholm University, Stockholm, Sweden; ^2^Department of Clinical Neuroscience, Karolinska Institutet, Stockholm, Sweden; ^3^Department of Behavioral Sciences and Learning, Linköping University, Linköping, Sweden

**Keywords:** attentional bias, attentional bias modification, social anxiety, virtual reality, dot-probe, attentional training

## Abstract

**Background:**

Attentional bias modification (ABM) aims to reduce anxiety by attenuating bias toward threatening information. The current study incorporated virtual reality (VR) technology and three-dimensional stimuli with a dot-probe task to evaluate the effects of a VR-based ABM training on attentional bias and anxiety symptoms.

**Methods:**

A total of 100 participants were randomized to four training groups. Attentional bias was assessed at pre- and post-training, and anxiety symptoms were assessed at pre-training, post-training, 1-week follow-up, and 3-months follow-up.

**Results:**

Change in anxiety did not correlate with change in bias (*p* = 0.24). A repeated-measures ANOVA showed no significant difference in bias from pre- to post-ABM (*p* = 0.144), or between groups (*p* = 0.976). For anxiety symptoms, a linear mixed-effects model analysis revealed a significant effect of time. Participants showed reduction in anxiety score at each successive assessment (*p* < 0.001). However, no other significant main effect or interactions were found. A clinically significant change analysis revealed that 9% of participants were classified as ‘recovered’ at 3-months follow-up.

**Conclusion:**

A single session of VR-based ABM did not change attentional bias. The significant reduction in anxiety was not specific to active training, and the majority of participants remained clinically unchanged.

## Introduction

Social anxiety disorder (SAD) is one of the most common mental health disorders, with an estimated prevalence rate of 3–13% in Western countries (Bandelow and Michaelis, 2015). SAD brings considerable distress to the individual, and has a negative impact on daily functioning, interpersonal relationships, and quality of life (Stein and Stein, 2008). While psychological and pharmacological treatments for SAD can be highly efficacious (see Mayo-Wilson et al., 2014; Curtiss et al., 2017), barriers such as low accessibility, high cost, time commitment, symptom of disorder (i.e., fear of interpersonal contact), and stigma have prevented many individuals from seeking treatment (Kessler et al., 2005; Dennis and O’Toole, 2014). Epidemiological studies on help-seeking behavior report that less than half of individuals suffering from anxiety disorders have sought professional help for their condition (e.g., Roness et al., 2005; Ruscio et al., 2008; Hunt and Eisenberg, 2010). Therefore, it is important to explore treatment options that are more effective, accessible, and acceptable for SAD patients (Heeren et al., 2015b). In recent years, attentional bias modification (ABM) has received considerable interest as a potential new treatment option.

Cognitive models of anxiety disorders posit that biased information processing plays a causal role in the development and maintenance of dysfunctional anxiety. In the context of SAD, attentional bias refers to the preferential allocation of attention toward socially threatening information (Bar-Haim et al., 2007; Cisler and Koster, 2010). A considerable body of literature has reported that clinically anxious individuals and healthy individuals with high trait anxiety exhibit threat-related attentional bias. These studies utilized a number of different assessment tasks, such as emotional Stroop (e.g., Andersson et al., 2006; Lee et al., 2009), spatial cuing (e.g., Fox et al., 2001), visual search (e.g., Rinck et al., 2003; De Voogd et al., 2014), and dot-probe (Macleod et al., 1986; Amir et al., 2008).

Attentional bias modification is grounded in the theory that anxiety vulnerability and symptoms can be attenuated by directly modifying the attentional bias implicated in the generation and maintenance of problematic anxiety (MacLeod et al., 2002; Koster et al., 2009; Bar-Haim, 2010; MacLeod and Mathews, 2012). Attentional modification is achieved through repeated training that results in an attentional shift away from threatening information (Cisler and Koster, 2010). One common way to design ABM programs is to introduce a training contingency to the task used to measure attentional bias. For instance, the training version of the dot-probe task has been used extensively to induce bias change (MacLeod and Mathews, 2012).

Despite numerous studies reporting that ABM reduces attentional bias and anxiety symptoms in both single-session (e.g., MacLeod et al., 2002; Amir et al., 2008) and multi-session (e.g., Dandeneau et al., 2007; Amir et al., 2009) training, the effectiveness of ABM as a therapeutic tool remains a contentious topic (Cristea et al., 2015, 2017; Mogg and Bradley, 2016; Grafton et al., 2017; Kruijt and Carlbring, 2018; McNally, 2018). Failure to replicate the bias and/or anxiety reduction after ABM training has been reported by several studies (e.g., Carlbring et al., 2012; Boettcher et al., 2013; McNally et al., 2013; Heeren et al., 2015a; Naim et al., 2018).

Meta-analyses have not managed to consolidate mixed findings from empirical studies to reach a consensus on whether ABM is a promising therapeutic tool. Researchers often draw very different conclusions from the results. For instance, both Linetzky et al.’s (2015) meta-analysis on ABM and Cristea et al.’s (2015) meta-analysis on cognitive bias modification (including both ABM and interpretive bias modification) estimated the overall effect size of bias modification on SAD to be around 0.40 (Cohen’s d/Hodges’ g). Linetzky and colleagues concluded that this was enough to support ABM as a ‘novel evidence-based treatment for anxiety disorders’ (p. 383). On the other hand, Cristea et al. pointed out that the effect sizes across the studies were small and highly heterogeneous. Removal of outliers significantly reduced overall effect size as well as heterogeneity, which suggests that publication bias may have inflated the estimated efficacy of ABM. The authors concluded that these results were not robust enough to support the use of ABM (and cognitive bias modification in general) as a treatment option for SAD.

One criticism of traditional ABM training is that the tasks can be quite repetitive. If the participant loses focus and fails to engage with the training fully, then the probability of bias/symptom change will also diminish (Heeren et al., 2015b). One potential strategy to increase engagement is the incorporation of new technology. Urech et al. (2015) conducted a proof of concept study, where ABM was carried out using virtual reality (VR) technology. The VR-based ABM successfully elicited bias change as well as a reduction in anxiety. There are several advantages associated with VR-based therapies. The extensive control that the experimenter has over the therapy environment and stimulus presentation ensures a consistent delivery of the treatment. The immersive nature of the VR environment and stimuli can potentially increase ecological validity and patient engagement. Furthermore, should VR-based ABM prove to deliver good clinical outcomes, the non-reliance on clinician coupled with increasing accessibility of VR programs could mean wider distribution and lower costs compared to treatment at a clinic (Lindner et al., 2017).

The aim of the current study was to test the effectiveness of a single-session, VR-based dot-probe task in reducing social anxiety in participants with elevated trait anxiety recruited from the general population. Firstly, we attempted to validate previous findings that dot-probe ABM training reduces attentional bias and anxiety symptoms. Secondly, we aimed to test whether ABM delivered in a virtual environment using three-dimensional (3D) facial expressions would increase the effectiveness of the training compared to regular two-dimensional (2D) stimuli. To this end, participants were assigned to one of four experimental groups receiving either active or mock ABM training with either 3D or 2D stimuli. Attentional bias was measured pre- and post-training using a 2D dot-probe task. Self-reported anxiety symptoms were assessed at pre-training, immediately post-training, at 1-week follow-up, and at 3-months follow-up. We hypothesized that, at post-training and follow-up assessments, (i) participants in the active ABM training groups will have significantly lower attentional bias and anxiety symptoms compared to those in the mock training groups; (ii) participants who received ABM with 3D stimuli will have lower attentional bias and anxiety symptoms compared to those who received ABM with 2D stimuli.

## Materials and Methods

### Participants

Recruitment took place between June and October, 2017. One hundred adult participants were recruited from the general population via advertisements on websites^1,2^, newspapers (Dagens Nyheter), and radio (Sveriges Radio P3). Potential participants were invited to visit the official study website iTerapi^3^ (Vlaescu et al., 2016), where they were provided with basic information about the study. Those who were interested in participating could then register an account to be screened.

Inclusion criteria were: (i) having a score of 30 or above on the Liebowitz Social Anxiety Scale, self-report (Fresco et al., 2001); (ii) normal depth perception; (iii) fluent Swedish speaker; and (iv) at least 18 years of age. Exclusion criteria were: (i) any psychological treatment/counseling within the past 90 days; (ii) any change in psychopharmacological medication within the past 90 days (with the exception of as-needed medications such as beta-blockers); and (iii) Depression and suicidal ideation [as indicated by a total score of 14 or higher, and/or a score greater than 0 on the suicide item of the Patient Health Questionnaire (Kroenke et al., 2001)]. The study was approved by the Regional Ethical Review Board in Stockholm, Sweden.

### Self-Reported Measures

The primary outcome measure was social anxiety assessed by the Liebowitz Social Anxiety Scale, self-report (LSAS-SR; Fresco et al., 2001). The LSAS-SR consists of 24 items, 13 of which are related to performance anxiety, while the remaining 11 are related to various social situations. The 24 items are first rated on a four-point Likert scale to indicate how much fear is associated with the situation described by each item. Then the same 24 items were rated again to indicate how much avoidance is associated with each situation. The LSAS-SR has good test–retest reliability, high structural validity, and internal consistency (Baker et al., 2002).

In addition to the LSAS-SR, participants also filled in several other questionnaires, including Patient Health Questionnaire (PHQ-9; Kroenke et al., 2001) for depression, Generalized Anxiety Disorder 7-item scale (GAD-7; Spitzer et al., 2006), the Difficulties in Emotion Regulation Scale-16 (DERS-16; Bjureberg et al., 2016), and Brunnsviken Brief Quality of Life Inventory (BBQ; Lindner et al., 2016). All measures were in Swedish. The DERS-16 and BBQ were originally developed in Swedish. Translated versions of the LSAS-SR, PHQ-9, and GAD-7 have all been validated and used in previous studies on clinical populations (e.g., Hansson et al., 2009; Hedman et al., 2010; Johansson et al., 2013).

### Attentional Bias Assessment and Modification Program

#### Apparatus

The VR program in which ABM took place was developed by Mimerse^4^. The Oculus Rift consumer version headset was used to run the program, and a wired Xbox 360 controller was used for response input. The VR program ran on a Corsair Tortuga computer with 4 Ghz Intel Core i7 processor and NVIDIA GeForce GTX 1080 graphics card.

#### Stimuli

The facial stimuli used in the current study were selected from the BP4D-Spontaneous Database (Zhang et al., 2014). A total of 32 individuals (50% female) each showing a neutral expression and a disgusted expression were included in the stimuli set, totaling to 64 expressions. Two sets of stimuli were created, with one set containing 2D images and one set containing 3D images. The stimuli sets were identical barring dimensionality. The 2D images have a resolution of 1040 × 1392 pixels.

Disgust was chosen as the socially threatening stimuli as it has been suggested that disgust closely relates to complex emotions that underpin social anxiety, such as shame, humiliation, and rejection (Phillips et al., 1998; Amir et al., 2003). It has been shown that individuals with social anxiety exhibit different patterns of brain activity when processing disgusted facial expression compared to non-anxious controls (Amir et al., 2005).

#### Dot-Probe Task

**FIGURE 1:**
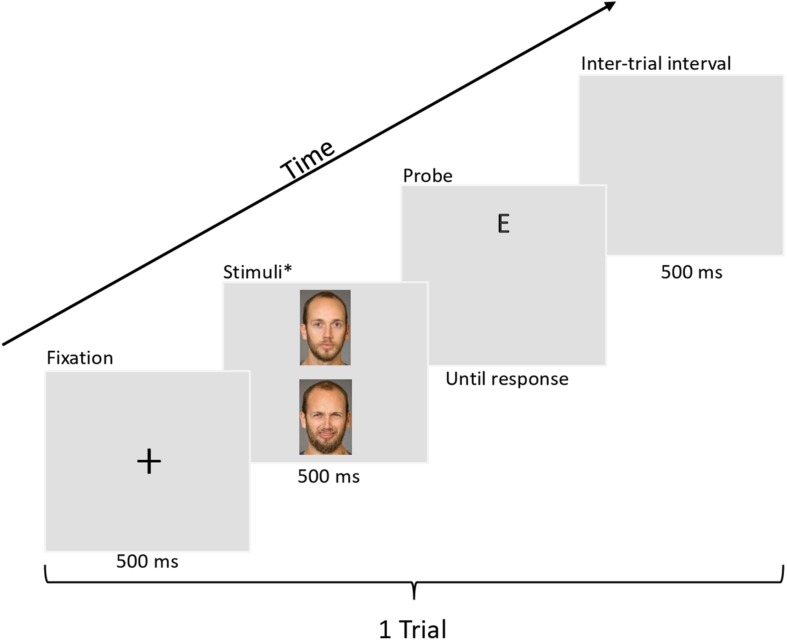
Example trial of a dot-probe task with 2D stimuli. The BP4D-Spontaneous database is proprietary, therefore the actual stimuli used are not permissible to print in publications. The faces shown in this example comes from the Umeå University Database of Facial Expressions (Samuelsson et al., 2012). All individuals in the database have provided written informed consent for their images to be used in research and publication. Full permission was granted to use these images by the database owner (P.C). Examples of the VR environment (as seen on a computer monitor) can be found in Supplementary Figure S1.

The dot-probe task was used to both measure and modify attentional bias. Each trial consisted of the following steps (see Figure 1): first, a fixation cross appeared in the middle of the screen for 500 ms. After the fixation cross, two faces appeared simultaneously (arranged vertically) on the screen for 500 ms. The two faces depicted the same individual showing a neutral expression and a disgusted expression. The position of the neutral/disgust expressions was counterbalanced, so that each expression appeared with equal frequency on top or bottom. After the faces disappeared, a probe (either letter ‘E’ or letter ‘F,’ with equal frequency) would appear in the location previously occupied by one of the facial expressions. The position of the probes was also counterbalanced so that they would appear equally frequently on top or bottom. Participants were instructed to identify the letter as quickly as possible by pushing the controller joystick left (for ‘E’) or right (for ‘F’). A 500 ms inter-trial interval took place before a new trial began.

#### Attentional Bias Measurement

In the measurement variant of the task, the probe appeared randomly in the location previously occupied by a neutral expression or a disgusted expression with equal frequency. Trials in which the probe appeared behind the disgusted expression are *congruent*. Trials in which the probe appeared behind the neutral expression are *incongruent*. A bias index was calculated by comparing a participant’s average reaction time in incongruent trials versus congruent trials.

Biasindex=Mean(RT)i⁢n⁢c⁢o⁢n⁢g⁢r⁢u⁢e⁢n⁢t-Mean(RT)c⁢o⁢n⁢g⁢r⁢u⁢e⁢n⁢t

A positive bias index thus indicated that the participant reacted faster to probes when they appeared behind disgusted faces, while a negative bias index indicated a faster reaction to probes behind neutral faces.

#### Active Training

The training variant of the dot-probe was exactly the same as the measurement variant with the addition of a training contingency – the probe always appeared in the location previously occupied by the neutral expression. This contingency is thought to modify bias by systematically directing the participant’s attention away from the threatening facial expressions. To conceal the training contingency, 20% of the trials across all conditions presented a neutral-neutral pairing, with the probe appearing randomly behind either faces.

#### Mock Training

Participants randomized to the mock groups received a mock training task lacking the training contingency (i.e., it was identical to the measurement task for the 2D mock group. For the 3D mock group, the training task was identical to the measurement task except that it was presented with 3D stimuli).

### Procedure

After registering and completing the screening questionnaires on iTerapi, participants eligible for the study were offered to book a time for their VR session at Stockholm University. Upon arrival, the participants first provided written informed consent to officially participate in the study. They then completed the pre-training assessment questionnaires (LSAS-SR, PHQ-9, GAD-7, DERS-16, and BBQ) on the iTerapi platform.

Group randomization was carried out by randomizing the VR session slots rather than the participants. Consecutive session slots were pseudorandomized in blocks of 4, 8, or 12 to the four experimental groups (2D mock training, 2D active training, 3D mock training, and 3D active training) using R. Since the order of group assignment was pre-designated, whenever a booked session was canceled, the group assignment for the slot (and subsequent slots) would transfer to the next session/participant. The VR data were linked to each participant by their unique participant ID from iTerapi. After confirming the participant’s iTerapi ID, the experimenter would manually enter the ID into the VR program before starting the experiment, thus linking each participant’s VR data to their questionnaire data. As the experimenters needed to manually input the correct code into the program script to initiate the assigned condition for each participant, they were not blind to the experimental conditions.

The VR session began with a brief visual acuity test using a Snellen chart inside the VR environment to ensure that all participants could see the images clearly. Next, the participants completed a dot-probe tutorial to familiarize themselves with the task. Upon successful completion the tutorial (five successive correct responses to probes), the participants completed 100 trials of measurement dot-probe to assess their baseline attentional bias pre-training. All measurement tasks were carried out using 2D stimuli, regardless of what stimuli were used in the training tasks. The participants then underwent two blocks of ABM training (190 trials each) with a self-paced break in between. Depending on their group affiliation, the participants received the following ABM during the training: (i) 2D mock: 380 trials of measurement dot-probe with 2D stimuli; (ii) 2D active: 380 trials of training dot-probe with 2D stimuli; (iii) 3D mock: 380 trials of measurement dot-probe with 3D stimuli; (iv) 3D active: 380 trials of training dot-probe with 3D stimuli. After the training phase, attentional bias was measured again using 100 trials of measurement dot-probe. Before leaving the lab, the participants filled in LSAS-SR to assess their anxiety post-training. Finally, participants were asked to complete the post-ABM assessment questionnaires (LSAS-SR, PHQ-9, GAD-7, DERS-16, and BBQ) on iTerapi at 7 and 90 days after their VR session. Reminder emails were sent to the participants along with the link to the questionnaires on the day of follow-up.

### Statistical Analyses

All statistical analyses were performed in R (version 3.5.2; R Core Team, 2018).

## Results

**FIGURE 2:**
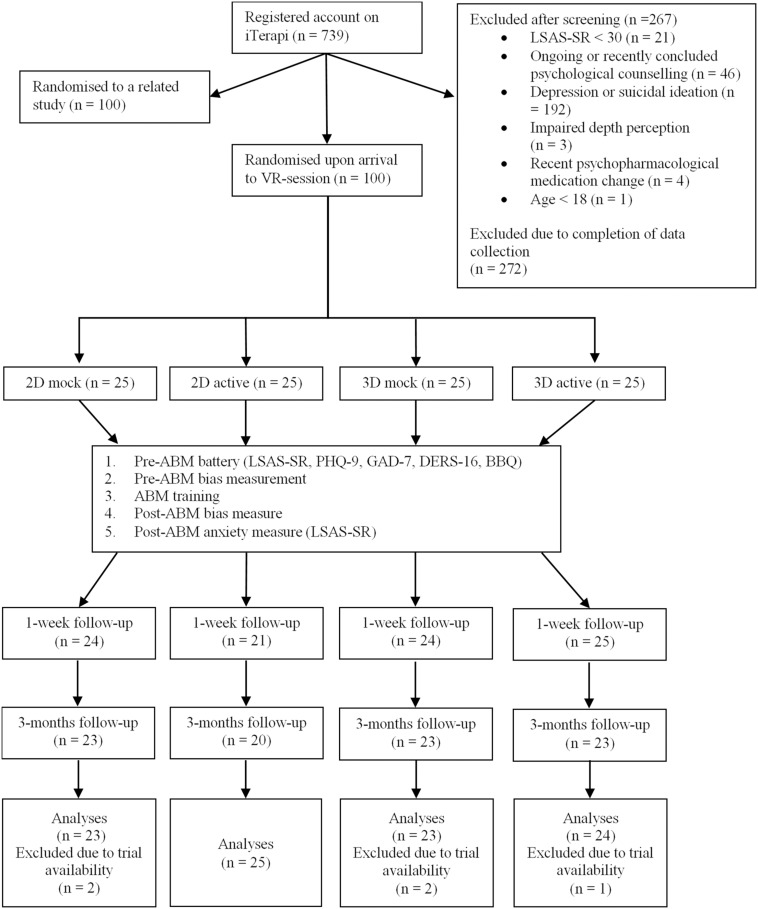
Overview of participant flow throughout the study.

All 100 participants completed pre-ABM and post-ABM assessment of anxiety and bias. For follow-up measures, six participants failed to complete the 1-week follow-up and 11 participants failed to complete the 3-months follow-up. For the bias measurement data, trials were discarded if they (i) were error trials; (ii) had a response time < 200 or > 2000 ms; or (iii) had a response time that was beyond 2 standard deviations from the individual’s mean response for each trial type (congruent/incongruent). Five participants had more than 20% of their trials discarded for at least one of the trial type in either the pre- or the post-training bias measurement task. These five participants were excluded from analyses (see Figure 2; for details of the data cleaning procedure, please refer to analysis script). Due to a technical error, the first trial of the first participant in each of the four groups failed to record. As a result, these four participants had a pre-ABM bias measurement with 99 trials instead of 100. None of the groups differ on any demographic characteristics or measures at baseline (see Table 1).

**TABLE 1:** Participant demographics and characteristics at baseline.

		**2D mock(*N* = 23)**	**2D active (*N* = 25)**	**3D mock (*N* = 23)**	**3D active (*N* = 24)**	**Between-groups comparison**
Female	*N (%)*	17(74%)	11(44%)	13(57%)	14(58%)	χ2 = 4.42 *p* = 0.219
Tertiary education	*N (%)*	16(70%)	12(48%)	14(61%)	13(54%)	χ2 = 2.51 *p* = 0.474
Age	*M (SD)*	40.70 (12.66)	40.72 (13.56)	38.48 (13.45)	43.29 (12.38)	*F*_(__3_,_91__)_ = 0.36, *p* = 0.782
Bias index	*M (SD)*	−6.07(32.94)	3.13 (29.72)	14.52 (43.22)	−4.45(39.17)	*F*_(__3_,_91__)_ = 0.92, *p* = 0.436
**Liebowitz Social Anxiety Scale, Self-reported**						
	*M (SD)*	71.00 (20.02)	68.68 (18.24)	70.35 (18.02)	69.00 (21.86)	*F*_(__3_,_91__)_ = 0.08, *p* = 0.973
**Patient Health Questionnaire**						
	*M (SD)*	5.65 (3.92)	5.56 (3.80)	4.96 (2.84)	5.04 (4.13)	*F*_(__3_,_91__)_ = 0.21, *p* = 0.886
**Generalized Anxiety Disorder 7-item scale**						
	*M (SD)*	5.43 (4.07)	5.32 (4.80)	6.09 (3.80)	5.58 (4.51)	*F*_(__3_,_91__)_ = 0.14, *p* = 0.934
**Difficulties in Emotion Regulation Scale-16**						
	*M (SD)*	40.52 (14.18)	36.80 (14.47)	43.26 (13.95)	38.50 (11.84)	*F*_(__3_,_91__)_ = 0.99, *p* = 0.402
**Brunnsviken Brief Quality of Life Inventory**						
	*M (SD)*	57.22 (20.22)	44.08 (24.36)	51.96 (19.51)	43.46 (15.76)	*F*_(__3_,_96__)_ = 2.51, *p* = 0.063

### Association Between Bias and Anxiety Symptoms

**FIGURE 3:**
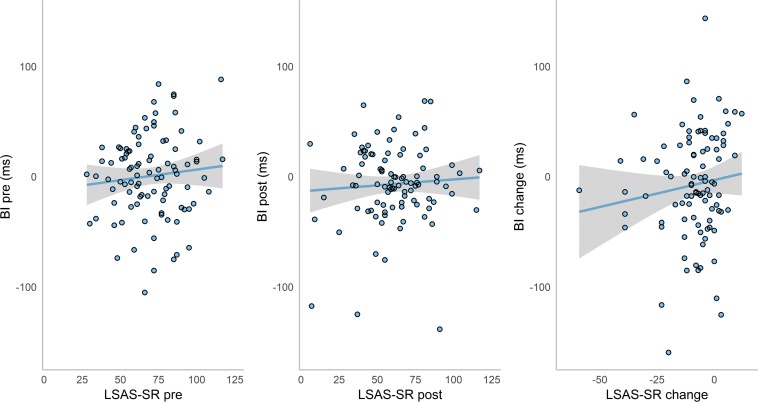
Scatterplots with trend lines of bias index (BI) and LSAS-SR scores at pre-ABM, post-ABM, and their change over time. Shaded regions indicate 95% CI.

Simple Pearson correlations were performed to investigate the relationship between attentional bias and anxiety symptoms. As shown in Figure 3 below, no correlation was found between bias index and LSAS-SR scores at pre-ABM (*r* = 0.10, *p* = 0.34) or post-ABM (*r* = 0.07, *p* = 0.52). Furthermore, no correlation was found between bias change and LSAS-SR change (*r* = 0.12, *p* = 0.24).

### Bias Change

To evaluate whether ABM training affected attentional bias, a 2 × 4 repeated measures ANOVA with the four groups as between-subjects factor and time (pre- vs. post-training) as within-subjects factor was performed. The results showed no significant difference in bias from pre- to post-training (*F*_(__1_,_91__)_ = 2.18, *p* = 0.144), or between groups (*F*_(__3_,_91__)_ = 0.07, *p* = 0.976). The interaction of group and time was also insignificant (*F*_(__3_,_91__)_ = 1.98, *p* = 0.122). This indicates that attentional bias did not change from pre- to post-training and that both observed bias and change in bias were not affected by group affiliation.

**FIGURE 4:**
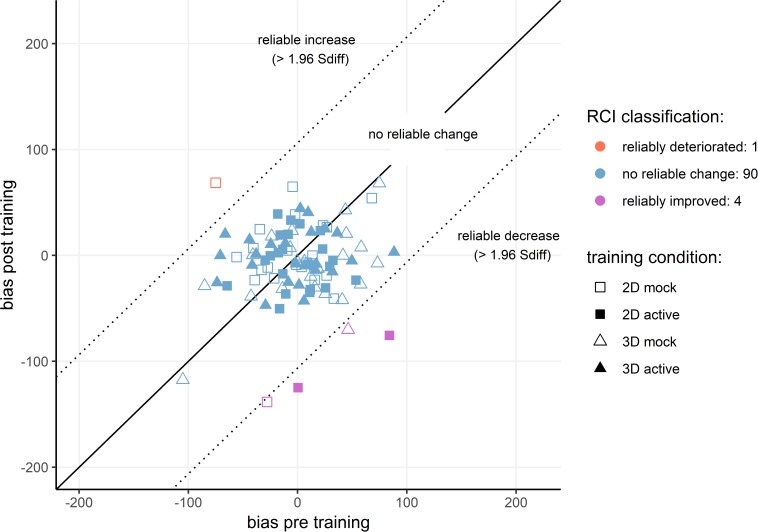
Reliable change plot for individual participant’s bias index.

To further explore achieved bias modification, we calculated reliable change indices for individual participant’s bias change (Jacobson and Truax, 1991). Note that for bias index we assessed only the first (reliable change) part of the dual-criterion calculation for clinical significant change proposed by Jacobson and Truax. First, the standard error of measurement (SE_M_) was calculated based on the sample baseline standard deviation and split-half reliability of the pre-training dot-probe task. The Spearman–Brown corrected average reliability estimate of 5000 random splits (Parsons et al., 2018) served as a measure of internal reliability for bias index. The resulting estimate was dramatically, but not uncharacteristically, low (*r* = −0.04). Then, a Standardized Difference Score (S_diff_) was computed based on the standard error of measurement (S_diff_ = √(2^∗^SE_M_^2^). For our sample, S_diff_ was 54.3 ms. If an individual’s bias index was reduced by at least 1.96 times the S_diff_, they were classified as showing a reliably improved bias (i.e., reduced preferential orienting toward threat). If an individual’s bias index increased by at least 1.96 times the S_diff_, they were classified as showing a reliably deteriorated bias toward negative. If an individual’s bias change fell within the range of 1.96 S_diff_, they were classified as unchanged. The results showed that one participant had a reliable deterioration in bias, 90 participants showed no reliable change, and only four participants achieved reliable improvement in their attentional bias after ABM training (Figure 4).

### Anxiety Change

To analyze and compare anxiety change between the four groups across time, a mixed model approach was used. Two dummy-coded variables replaced the group variable to better dissociate the effects of training condition (mock = 0 vs. active = 1) and stimuli used (2D = 0 vs. 3D = 1). We used the nlme() package (Pinheiro et al., 2018) in R to compare different models on their fit to the data using the Akaike information criterion (AIC). Various models outperformed the null model, with AIC values ranging from 2969.3 (effects of time and condition, plus their interaction) to 3302.8 (null model with intercept only). For details of model comparison, see Supplementary Table S1. Given that comparing the effects of 3D versus 2D stimuli was a key interest in the current study, we present the results from the full model first. Following that, we will also outline the results from the simpler time and condition interaction model, which had the lowest AIC value but did not have a significantly better fit than the full model (likelihood-ratio = 3.43, *p* = 0.489).

**FIGURE 5:**
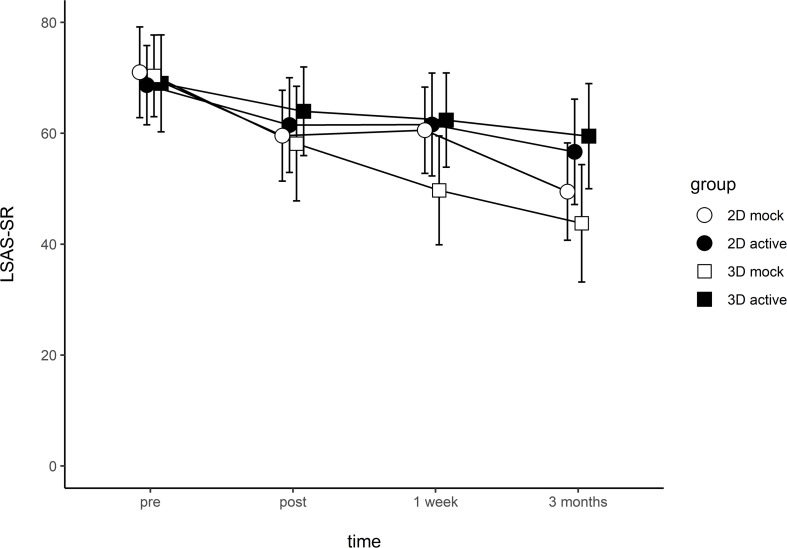
Liebowitz Social Anxiety Scale, Self-reported (LSAS-SR) score change across time. Error bars represent 95% CI.

A linear mixed-effects model analysis was carried out using the full model. Time, condition, 2D/3D stimuli, and all two-way and three-way interactions were modeled as fixed effects. Random intercepts and random slopes for each participant were modeled as random effects. For main effects, only time was significant – on average, participants showed a reduction of 5.9 points in their LSAS-SR score at each successive assessment (*t*_(__266__)_ = −5.31, *p* < 0.001). No other main effects or interactions were significant (Table 2). Figure 5 illustrates the LSAS-SR reduction over time, separated by groups.

**TABLE 2:** Fixed effects parameter estimates.

**Effect**	**Estimate**	***SE***	**DF**	***t***	***p***
*Intercept*	75.12	4.66	266	16.13	< 0.001^***^
*Time*	–5.86	1.10	266	–5.31	< 0.001^***^
*Condition*	–5.06	6.47	91	–0.78	0.44
*2D/3D*	1.94	6.59	91	0.29	0.77
*Time × condition*	2.86	1.56	266	1.83	0.07
*Time × 2D/3D*	–2.67	1.56	266	–1.71	0.09
*Condition × 2D/3D*	–0.60	9.18	91	–0.07	0.95
*Time × condition × 2D/3D*	2.57	2.19	266	1.17	0.24

**^∗∗∗^p* < 0.001. SE, standard error; DF, degrees of freedom.*

The simpler model with the lowest AIC value only had time, condition, and their interaction as fixed effects. Again, random intercepts and random slopes for each participant were modeled as random effects. In this model, the main effect of time was significant (*t*_(__268__)_ = −9.13, *p* < 0.001), but the main effect of condition was not (*t*_(__93__)_ = −1.18, *p* = 0.242). There was also a significant interaction between time and condition (*t*_(__266__)_ = −3.75, *p* < 0.001), indicating higher average anxiety reduction in the mock training groups compared to the active training groups. Directly comparing the simple model against the full model did not reveal a significantly better fit.

### Clinically Significant Change

Jacobson-Truax clinical change indices were computed for LSAS-SR scores. Here we applied the full clinical change index calculation (as opposed to just the reliable change calculation done for bias change). For each participant, reliable change was determined first (defined as change surpassing 1.96 S_diff_), followed by application of the A criterion to determine clinical change. The A criterion was based on the sample baseline distribution of LSAS-SR scores – participants whose post-training scores were lower than the baseline group mean score minus 1.96 times the baseline standard deviation were classified as ‘recovered,’ indicating that their post-ABM scores fall outside the 95% confidence interval of the sample’s distribution at baseline. The resulting cut-off score for this sample was 32, which happens to be quite close to the generally defined LSAS-SR cut-off score of 30, below which SAD is considered unlikely.

For the calculation of the reliable change criterion, Cronbach’s alpha was determined using the psych package (Revelle, 2018). The internal reliability of the LSAS-SR was found to be satisfactory (α = 0.92), and the resulting S_diff_ was 7.7 points. Scores at post-training, 1-week, and 3-months follow-ups were all compared to baseline. At each time point, participants who showed reliable change (i.e., changed more than 1.96 S_diff_) and a score below 32 were classified as ‘recovered.’ Participants who scored below 32 but did not show reliable change were classified as ‘non-reliably recovered.’ Participants who showed reliable change but did not score below 32 points were classified as ‘improved.’ Participants who did not show reliable change were classified as ‘unchanged.’ Participants who showed reliable increase in LSAS-SR scores would have been classified as ‘deteriorated.’

**FIGURE 6:**
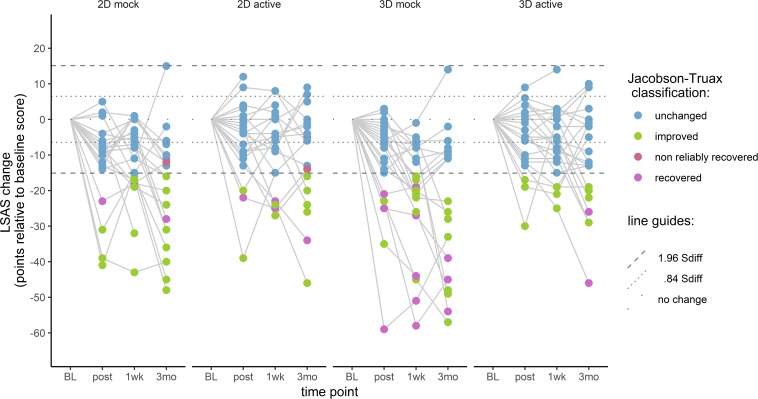
Jacobson-Truax (Criterion A) classification of individual LSAS-SR change across time. BL, baseline; 1wk, 1-week follow-up; 3mo, 3-months follow-up.

At the 3-months follow-up, eight participants were classified as ‘recovered,’ five ‘non-reliably recovered,’ 29 ‘improved,’ and 43 ‘unchanged.’ When looking at individual changes by group (Figure 6), it appears that participants in the mock training groups achieved ‘recovered’ or ‘improved’ status more often than those in the active training groups.

### Secondary Outcome Measures

All secondary outcomes were analyzed using linear mixed models in R. No significant changes were observed for PHQ-9, GAD-7, and BBQ, indicating that the ABM training had no effect on depression, generalized anxiety, or quality of life. For emotional regulation, there was a significant interaction of time and stimuli. On average, participants in the 3D stimuli groups scored 2.3 points lower on the DERS-16 at each time point post-training compared to those in the 2D groups (*t*_(__168__)_ = −2.35, *p* = 0.020). This reduction indicated increased emotional regulation post-ABM, and that 3D stimuli were associated with better improvements (a summary of all score changes over time can be found in Supplementary Table S2).

## Discussion

The current study investigated the efficacy of a VR-based ABM program in reducing social anxiety in participants with LSAS-SR scores comparable to a clinical population. After a single session of ABM training, we did not observe any changes in attentional bias. In terms of anxiety symptoms, participants reported lower LSAS-SR scores compared to the baseline. This reduction was maintained at the 1-week and 3-months follow-ups. Contrary to our hypothesis, all groups showed reduction in anxiety symptoms post-training, regardless of training contingency. Also contrary to our hypothesis, 3D stimuli did not result in better anxiety reduction than 2D stimuli. At the 3-months follow-up, around 9% of participants met the criteria for clinically significant change, while half of the participants remained unchanged.

### Failure to Detect Bias

The current study adds to a number of previous reports in finding no evidence of attentional bias in socially anxious individuals at baseline (e.g., Julian et al., 2012; Boettcher et al., 2013; Badura-Brack et al., 2015; Heeren et al., 2015a; Miloff et al., 2015; Naim et al., 2018; for a recent meta-analysis of baseline bias in ABM RCTs, see Kruijt et al., 2019). Furthermore, we failed to detect a change in attentional bias post-ABM training. Proponents of ABM have argued that if a *procedure* (i.e., dot-probe task) fails to modify attentional bias, then such a procedure cannot reveal the true effect of a successful ABM *process* on anxiety (MacLeod and Grafton, 2016). One fundamental problem that plagues the ABM field is the lack of reliable measure for attentional bias (Rodebaugh et al., 2016). Despite being one the most commonly used method to assess and measure attentional bias, it has been pointed out that the dot-probe task does not possess sufficient reliability to reveal individual differences in attentional bias (e.g., Schmukle, 2005; Waechter and Stolz, 2015; Chapman et al., 2017). In line with these concerns, the internal reliability of the bias indices derived from our dot-probe data was also low (Spearman–Brown corrected split-half estimate = −0.04). We argue that the most pressing concern of ABM research is to develop methods that can reliably measure attentional bias, as any attempt at developing an efficacious ABM procedure would be futile without a reliable bias estimate. Nevertheless, we acknowledge the theoretical importance of distinguishing between process and procedure, thus limiting further discussion of the results to the procedures used, rather than ABM process in general.

### Symptom Reductions

Despite the failure to detect or change bias, our data showed that anxiety scores decreased relatively consistently across all groups. This symptom reduction is maintained at the 3-months follow-up, albeit only a small percentage (9%) of participants achieved clinically significant change. A number of studies have reported that both active ABM and mock ABM can induce similar levels of symptom reduction (e.g., Boettcher et al., 2013; Bunnell et al., 2013; McNally et al., 2013; Enock et al., 2014). These findings suggest that while the ABM procedures failed to measure/change attentional bias, they might still deliver some therapeutic effect. However, the mechanism of this symptom reduction is unclear. In our study, the observed symptom change cannot be attributed to the hypothesized mechanism of action (i.e., the presence of a training contingency). The anxiety reduction could be due to any number of non-specific treatment factors. For instance, placebo effect resulted from being selected to take part in a study involving novel treatment for social anxiety could lead to symptom reduction (Enock et al., 2014). It has also been proposed that mere attention training without contingency is sufficient for anxiety reduction (Heeren et al., 2015a).

While the full model used in our main mixed-effects model analysis did not reveal any significant differences between the active ABM training condition and the mock training condition, it is worth noting that a simpler model with only the factors time and condition (i.e., aggregating 2D and 3D training groups) did reveal a significant interaction, indicating that mock training outperformed active training in anxiety reduction. Such findings have been reported previously. Badura-Brack et al. (2015) conducted two separate randomized controlled trials where patients suffering from post-traumatic stress disorder (PTSD) underwent dot-probe training. In both trials, participants who were in the mock condition showed more symptom reduction compared to those who were in the active condition. The authors attributed the superior performance of the mock dot-probe task to it being a better attentional control task – since the probes appeared behind threatening and neutral stimuli equally frequently, the participants implicitly learned to ignore the threatening stimuli to complete the task efficiently. However, we stress that this model did not have a significantly better fit than the full model, and it did not account for a major experimental factor in the study design – namely the comparison of 2D versus 3D stimuli.

In addition to the LSAS-SR, we also observed significant reductions in DERS-16 scores across all groups, indicating improved emotional regulation. The improvements in secondary outcome measures are supportive of the notion that non-specific treatment effects shared by active ABM and mock ABM may offer therapeutic value without influencing attentional bias. No further significant effects were observed for either DERS-16 or any other secondary outcome measures [depression (PHQ-9), generalized anxiety (GAD-7), and quality of life (BBQ)].

### VR Technology

To our knowledge, this was the first study that employed three-dimensional facial expressions as stimuli in a dot-probe ABM task. While the LSAS-SR data did not reveal a significant effect of stimuli, emotional regulation (as measured by DERS-16) improved more with 3D stimuli. It is possible that the novelty factor of VR-technology and 3D stimuli increased engagement to the ABM task, thus enhancing whatever therapeutic effects delivered. Unfortunately, without a clear understanding of the underlying mechanisms, it is difficult to substantiate such a claim. Nevertheless, how 3D stimuli interact with VR-based psychological therapy is an interesting topic in itself and worthy of further investigation.

### Limitations

The results from the current study should be interpreted in light of a number limitations. Firstly, despite the average LSAS-SR score at baseline (70) indicated probable SAD diagnosis (Rytwinski et al., 2009), all symptom measures were self-reported without formal, clinician-administered diagnostic assessments. It has been suggested that attentional bias measures from non-clinical populations could be more inconsistent compared to clinical ones (Mogg et al., 2000; Schmukle, 2005), although data supporting such a claim are scarce. Furthermore, our exclusion criterion only pertains to recent changes in psychopharmacological medication. As we did not gather data on existing medication use, it was not possible to see whether anxiolytic medication affected the results. Secondly, the facial expressions used in the current study were not validated. Indeed, during data collection, experimenters noted how participants occasionally referred to the negative facial expressions as ‘angry’ instead of ‘disgusted.’ Thirdly, since the current study lacks a wait list control group, it is difficult to discern whether symptom reduction was ABM-specific, or due to factors such as spontaneous recovery or regression to the mean. At least one study that included a wait list control group reported that both ABM and mock ABM are superior to wait list (e.g., Enock et al., 2014). Lastly, while the initial viewing distance inside the VR was fixed at the start of the experiment, participants could adjust viewing distance freely by leaning forward or backward. As a result, the viewing experience of individual participants could vary. We ensured that all participants could see the images clearly by administering a brief visual acuity test using a Snellen chart inside the VR environment prior to starting the experiment, and randomization to different groups should also control for the effects introduced by subjective viewing experience.

The use of a single-session ABM in the current study could be seen as a limitation, as it makes intuitive sense to expect a more pronounced effect following multiple sessions of training. However, recent meta-analyses that assessed the effect of ABM dosage did not provide evidence in support of such a ‘more is better’ notion. For instance, Cristea et al.’s (2015) meta-analysis on cognitive bias modification found a slightly negative linear relationship between effect sizes for general anxiety and the number of training sessions. Similarly, Price et al. (2017) reported a paradoxical finding that high dosage of ABM training did not outperform controls, whereas fewer number of trials resulted in significant anxiety reduction. Heeren et al.’s (2015b) meta-analysis reported that neither the number of training sessions nor the number of training trials were a significant moderator of ABM effects on SAD symptoms. As a result, we feel that our use of a single-session training is justified.

## Conclusion

A single-session, VR-based dot-probe ABM training revealed no significant differences between active and mock training. A significant reduction in anxiety was observed in both conditions, which was maintained at 3-months follow-up. The anxiety reduction could not be attributed to changes in attentional bias, as we failed to detect bias at baseline, nor could we change bias with ABM training. In this first attempt at comparing ABM using 2D versus 3D stimuli, we did not observe any differences in anxiety or bias. It was found that groups receiving 3D training showed more improvement on a secondary outcome measure of emotional regulation. We believe that the effects of 3D stimuli, along with the use of VR technology in psychological treatments, warrant further investigation. The current study indicated no substantial treatment gains from ABM, as symptom reduction appeared to be non-specific. More accurate, reliable, and precise measures of attentional bias are needed before we can properly assess the efficacy of any ABM procedure.

## Open Science and Pre-Registration

We strive to adhere to the principles of Open Science. Unfortunately, the current study was not pre-registered before the commencement of data collection. In our effort to best compensate for the lack of pre-registration, all data generated in the current study, as well as the complete R script used for data cleaning and analyses will be made openly accessible (see Data Availability Statement below).

## Data Availability Statement

All datasets generated for this study can be found online at: https://doi.org/10.17045/sthlmuni.7813439.

## Ethics Statement

This study was carried out in accordance with the recommendations of The Swedish Ethical Review Authority with written informed consent from all subjects. All subjects gave written informed consent in accordance with the Declaration of Helsinki. The protocol was approved by the Regional Ethical Review Board in Stockholm, Sweden.

## Author Contributions

LM and PC contributed conception and design of the study. GA provided the participant recruitment and data collection platform. LM, SN, and EZ collected and organized the data. LM and A-WK performed the statistical analyses. LM wrote the manuscript. A-WK, PC, and GA contributed to manuscript revision. All authors read and approved the submitted version.

## Conflict of Interest

The authors declare that the research was conducted in the absence of any commercial or financial relationships that could be construed as a potential conflict of interest.

## Footnotes

1 http://studie.nu

2 http://facebook.com

3 http://iterapi.se

4 https://mimerse.com
