# Supplementary material for: Attentional Bias Modification in Virtual Reality – A VR-Based Dot-Probe Task With 2D and 3D Stimuli
Source: Front Psychol. 2019 Nov 13;10:2526. doi: 10.3389/fpsyg.2019.02526 (PMC6863810; doi:10.3389/fpsyg.2019.02526)
Supplement: Supplementary file 1 [file Image_1.pdf]

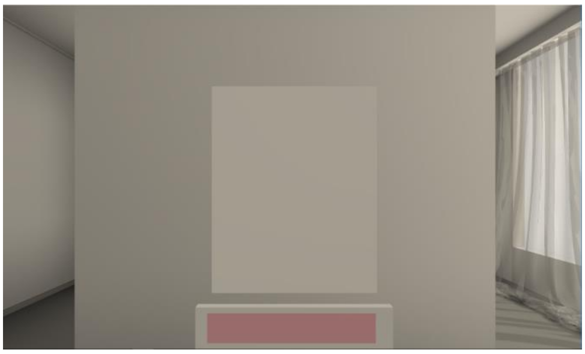

(a) VR environment

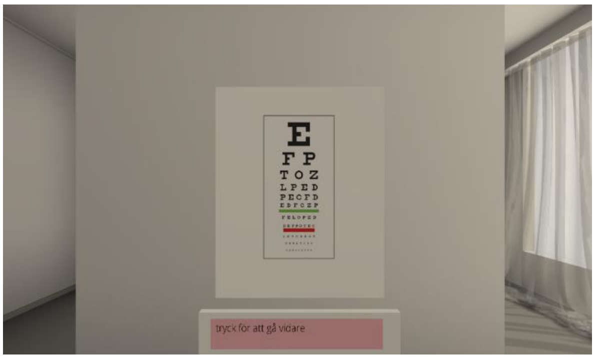

(b) Snellen chart for visual acuity check

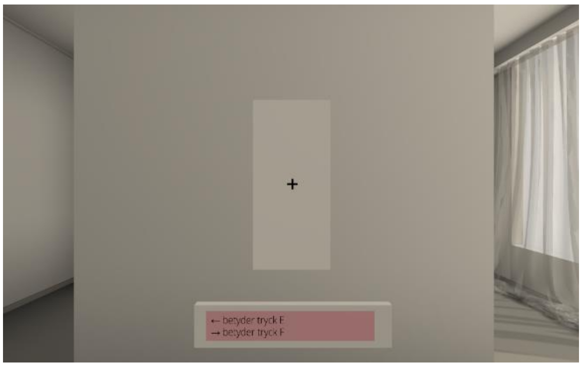

(c) Fixation cross

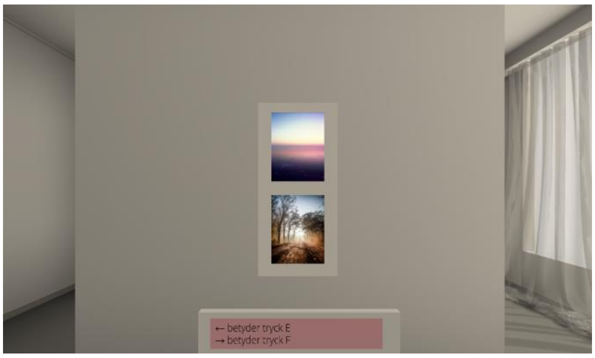

(d) Stimuli presentation

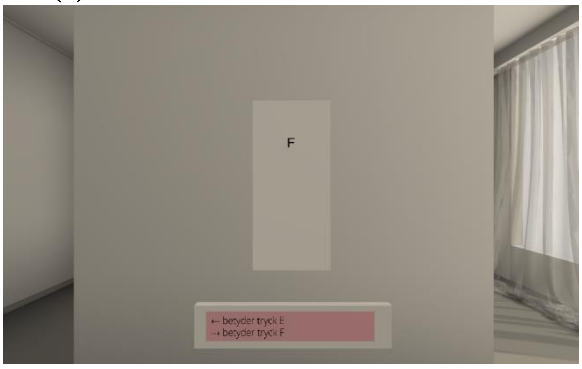

(e) Probe presentation

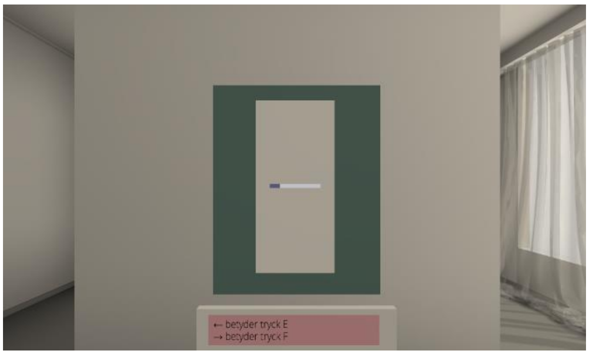

(f) Response feedback (tutorial only)

Figure S1. Screenshots of the virtual environment.
