# Supplementary material for: Attentional Bias Modification in Virtual Reality – A VR-Based Dot-Probe Task With 2D and 3D Stimuli
Source: Front Psychol. 2019 Nov 13;10:2526. doi: 10.3389/fpsyg.2019.02526 (PMC6863810; doi:10.3389/fpsyg.2019.02526)
Supplement: Supplementary file 2 [file Table_1.DOCX]

*Mixed-Effects Model Comparison*

| **Model** | **Model component** | | | **AIC** |
| --- | --- | --- | --- | --- |
|  | **Main effect** | **Interaction** | **Random effect**  **(participant)** |  |
| 1 | intercept only (null model) | - | - | 3302.8 |
| 2 | null model | - | intercept | 3082.1 |
| 3 | null model | - | intercept and slope | 3031.9 |
| 4 | time | - | intercept and slope | 2979.3 |
| 5 | condition | - | intercept and slope | 3033.4 |
| 6 | time + condition | time * condition | intercept and slope | **2969.3** |
| 7 | time + condition + stimuli | all 2-way interactions | intercept and slope | 2973.3 |
| 8 | time + condition + stimuli | all 2-way interactions and 3-way interaction | intercept and slope | **2973.9** |

**Note.* Model 1 was fitted with generalised least squares (gls) with maximum likelihood (ML). Models 2-8 were fitted with linear mixed-effects (lme) with ML.
