# Supplementary material for: Attentional Bias Modification in Virtual Reality – A VR-Based Dot-Probe Task With 2D and 3D Stimuli
Source: Front Psychol. 2019 Nov 13;10:2526. doi: 10.3389/fpsyg.2019.02526 (PMC6863810; doi:10.3389/fpsyg.2019.02526)
Supplement: Supplementary file 3 [file Table_2.DOCX]

*Summary of Self-Reported Measures Across Time, Separated by Groups*

|  | **Group** | **2D active**  ***M (SD)*** | **2D mock**  ***M (SD)*** | **3D active**  ***M (SD)*** | **3D mock**  ***M (SD)*** |
| --- | --- | --- | --- | --- | --- |
|  | ***N*** | **25** | **23** | **24** | **23** |
| ***Liebowitz* *Social* *Anxiety* *Scale*, *Self*-*reported*** | | | | | |
| Pre |  | 68.68 (18.24) | 71.00 (20.02) | 69.00 (21.86) | 70.35 (18.02) |
| Post |  | 61.48 (21.79) | 59.57 (20.06) | 63.96 (19.98) | 58.13 (25.29) |
| 1-week |  | 61.57 (21.70) | 60.55 (18.61) | 62.38 (21.25) | 49.70 (24.03) |
| 3-months |  | 56.65 (21.67) | 49.48 (20.51) | 59.48 (23.18) | 43.76 (24.77) |
| ***Patient* *Health* *Questionnaire*** | | | | | |
| Pre |  | 5.56 (3.80) | 5.65 (3.92) | 5.04 (4.13) | 4.96 (2.84) |
| 1-week |  | 5.52 (4.93) | 5.23 (3.98) | 5.04 (4.33) | 3.78 (2.49) |
| 3-months |  | 6.55 (6.02) | 4.70 (3.56) | 4.26 (3.39) | 4.60 (3.27) |
| ***Generalised* *Anxiety* *Disorder* 7-*item* *scale*** | | | | | |
| Pre |  | 5.32 (4.80) | 5.43 (4.07) | 5.58 (4.51) | 6.09 (3.80) |
| 1-week |  | 3.76 (4.01) | 4.86 (4.05) | 5.12 (4.40) | 3.57 (3.00) |
| 3-months |  | 4.05 (3.61) | 5.10 (4.19) | 4.74 (3.78) | 4.60 (2.80) |
| ***Difficulties* *in* *Emotion* *Regulation* *Scale*-*16*** | | | | | |
| Pre |  | 36.80 (14.47) | 40.52 (14.18) | 38.50 (11.84) | 43.26 (13.95) |
| 1-week |  | 34.00 (15.24) | 35.33 (13.38) | 38.42 (11.85) | 36.83 (14.27) |
| 3-months |  | 35.00 (15.59) | 35.55 (12.97) | 35.35 (12.16) | 30.45 (11.58) |
| ***Brunnsviken* *Brief* *Quality* *of* *Life* *Inventory*** | | | | | |
| Pre |  | 44.08 (24.36) | 57.22 (20.22) | 43.46 (15.76) | 51.96 (19.51) |
| 1-week |  | 42.76 (24.85) | 55.71 (19.50) | 46.96 (14.89) | 56.61 (20.03) |
| 3-months |  | 40.70 (24.53) | 56.95 (18.61) | 45.96 (14.21) | 54.40 (22.10) |
